# In memoriam of Anna D Polityko (17.12.1959 — 20.04.2013)

**DOI:** 10.1186/1755-8166-7-2

**Published:** 2014-01-14

**Authors:** Ivan Iourov, Yuri Yurov, Henry Heng, Thomas Liehr

**Affiliations:** 1 National Research Center of Mental Health, Russian Academy of Medical Sciences, Moscow, Russia; 2 Wayne State University School of Medicine, 540 E Canfield Street, Detroit, MI 48201, USA; 3 Jena University Hospital, Institute of Human Genetics, Friedrich Schiller University, Jena, Germany

## 

**Figure:**
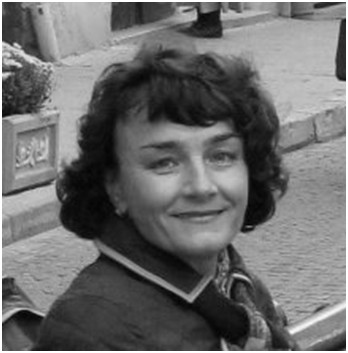
Professor Anna D Polityko

Last year, the Editorial Board of *Molecular Cytogenetics* had to record with the deepest regret the passing of one of its members after a long and courageous battle with cancer — Professor Anna D Polityko. She was a renowned specialist in cytogenetics, a Senior Researcher at the National Medical Center “Mother and child”, and the International Sakharov Environmental University (Minsk, Belarus), and the Belarus representative for the European Advisory Council of the ECA (European Cytogenetic Association). Moreover, being an active member of the *Molecular Cytogenetics* Editorial Board, she has significantly contributed to the recognized success of the journal.

Anna D Polityko was born on December 17, 1959 in Glukhov (formely USSR; nowadays it is a part of Ukraine). In 1982, she graduated from Belarusian State University (Minsk, Belarus) and started her career at the Institute of Hereditary Diseases of the Belarusian Ministry of Health (Minsk, Belarus). In 1998, she finished her PhD thesis on “Cytogenetic characteristics and *in vitro* radiosensitivity in persons with balanced constitutional karyotypic abnormalities”. As a qualified specialist, Anna provided consulting services in regional genetics centers in Belarus, peformed diagnosis of chromosome-related diseases, and controlled the quality and efficiency of the latter. She contributed a lot to the fields of cytogenetics and molecular cytogenetics, being involved in numerous national and international research projects. In recognized scientific databases, more than 20 articles with her co-authorship can be found, excluding those 248 publications published in Russian.

On behalf of the Editorial Board, we wish to express our deepest sorrow to her family, including two children and one granddaughter, and to all her friends and colleagues. We all have lost a highly motivated colleague full of brilliant ideas, a key person in the improvement of cytogenetic/molecular cytogenetic diagnosis in her country and more importantly a very kind and supporting friend. Anna will be remembered as an exceptionally strong, enthusiastic and optimistic person. We miss her sorely.

